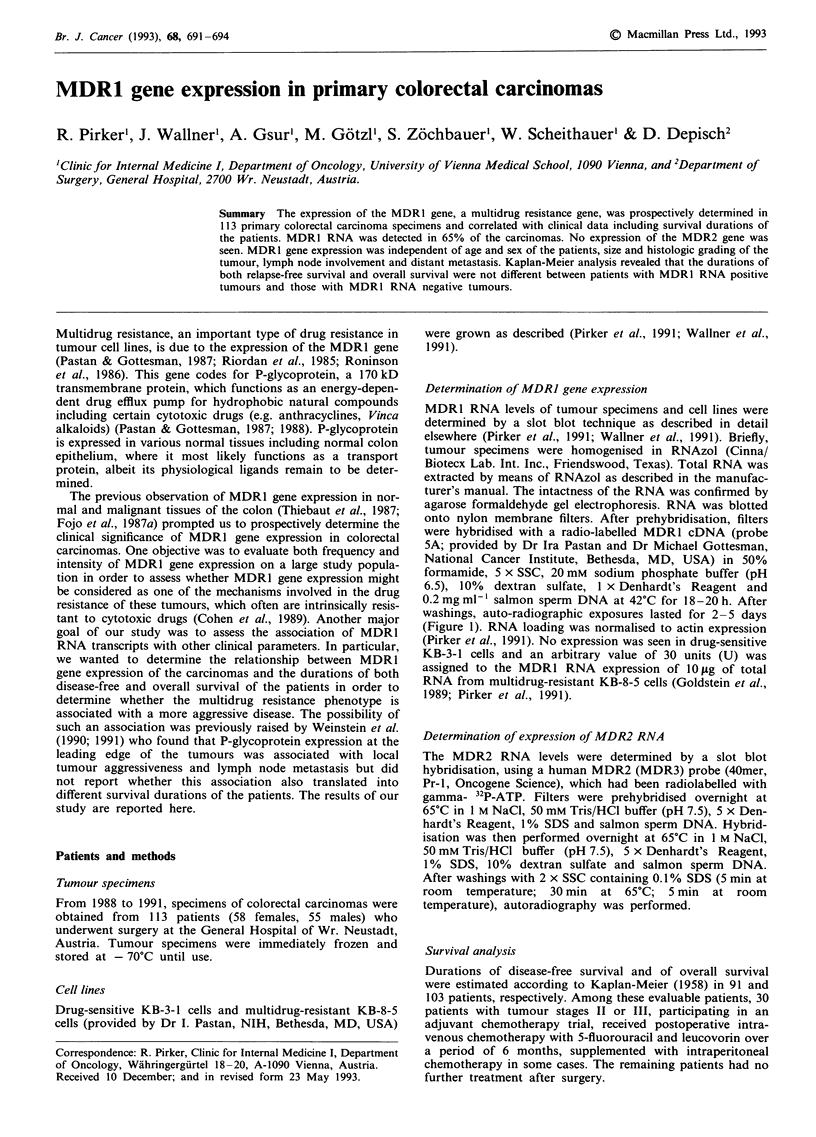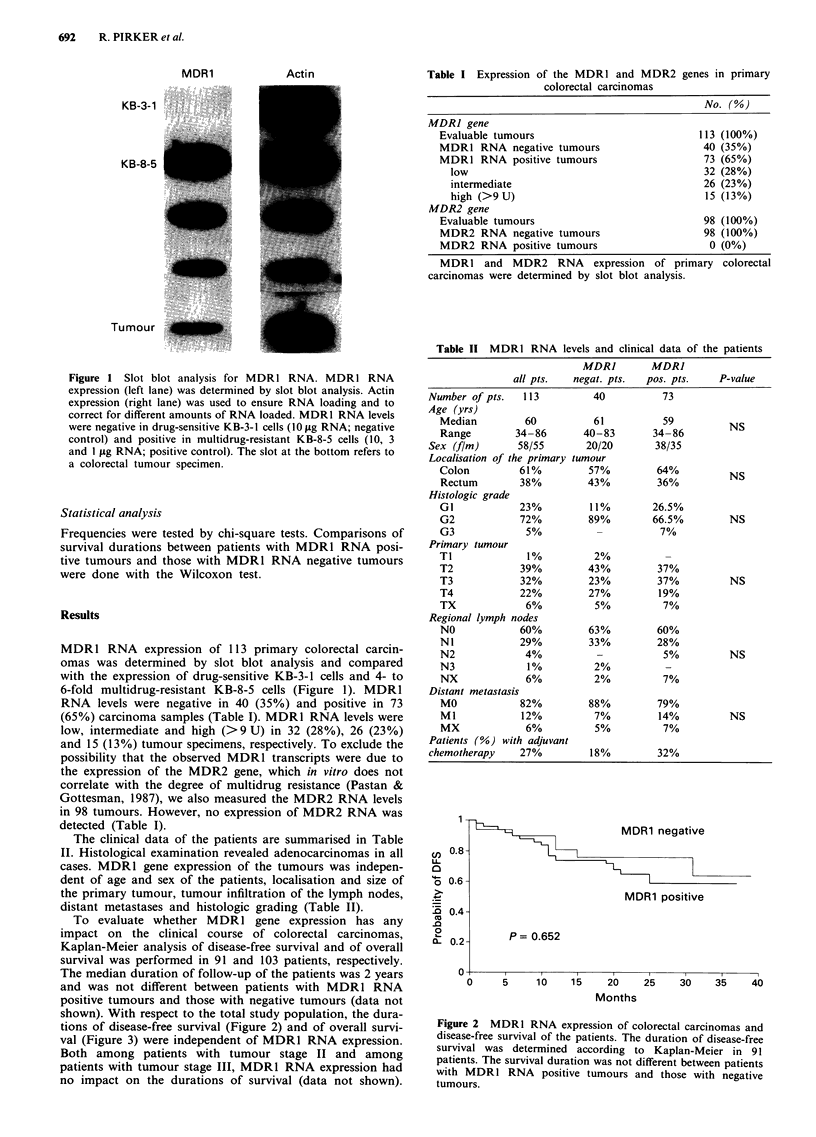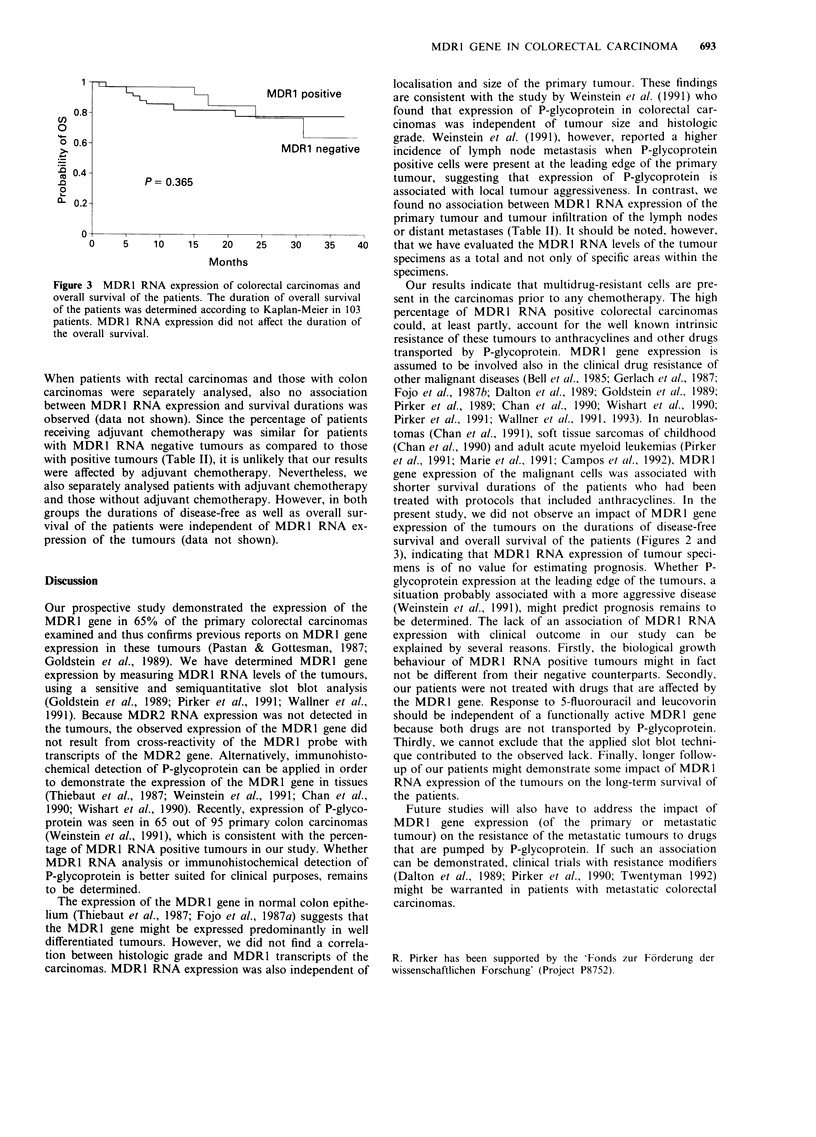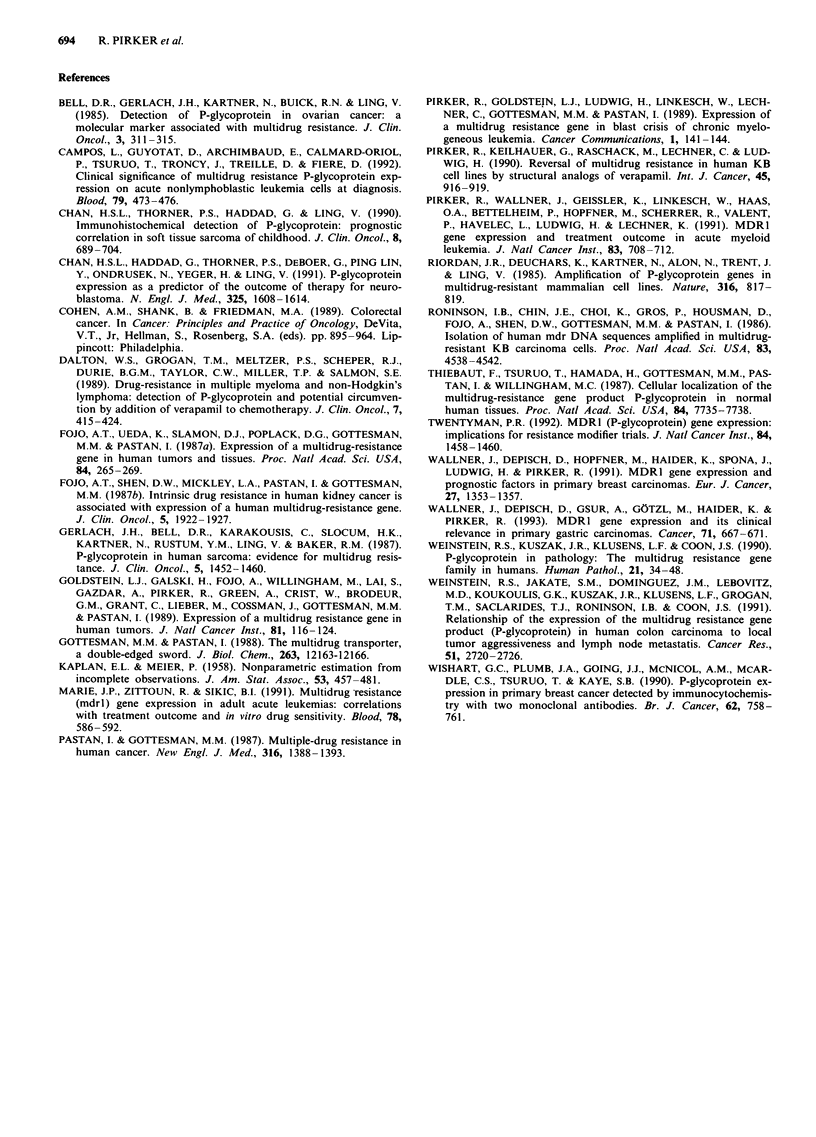# MDR1 gene expression in primary colorectal carcinomas.

**DOI:** 10.1038/bjc.1993.411

**Published:** 1993-10

**Authors:** R. Pirker, J. Wallner, A. Gsur, M. Götzl, S. Zöchbauer, W. Scheithauer, D. Depisch

**Affiliations:** Department of Oncology, University of Vienna Medical School, Austria.

## Abstract

**Images:**


Br. J. Cancer (1993), 68, 691–694 © Macmillan Press Ltd., 1993

### MDR1 gene expression in primary colorectal carcinomas

R. Pirker[1], J. Wallner[1], A. Gsur[1], M. Götzl[1], S. Zöchbauer[1], W. Scheithauer[1] & D. Depisch[2]

[1]*Clinic for Internal Medicine I, Department of Oncology, University of Vienna Medical School, 1090 Vienna, and* [2]*Department of Surgery, General Hospital, 2700 Wr. Neustadt, Austria.*

**Summary** The expression of the MDR1 gene, a multidrug resistance gene, was prospectively determined in 113 primary colorectal carcinoma specimens and correlated with clinical data including survival durations of the patients. MDR1 RNA was detected in 65% of the carcinomas. No expression of the MDR2 gene was seen. MDR1 gene expression was independent of age and sex of the patients, size and histologic grading of the tumour, lymph node involvement and distant metastasis. Kaplan-Meier analysis revealed that the durations of both relapse-free survival and overall survival were not different between patients with MDR1 RNA positive tumours and those with MDR1 RNA negative tumours.

Multidrug resistance, an important type of drug resistance in tumour cell lines, is due to the expression of the MDR1 gene (Pastan & Gottesman, 1987; Riordan *et al.*, 1985; Roninson *et al.*, 1986). This gene codes for P-glycoprotein, a 170 kD transmembrane protein, which functions as an energy-dependent drug efflux pump for hydrophobic natural compounds including certain cytotoxic drugs (e.g. anthracyclines, *Vinca* alkaloids) (Pastan & Gottesman, 1987; 1988). P-glycoprotein is expressed in various normal tissues including normal colon epithelium, where it most likely functions as a transport protein, albeit its physiological ligands remain to be determined.

The previous observation of MDR1 gene expression in normal and malignant tissues of the colon (Thiebaut *et al.*, 1987; Fojo *et al.*, 1987*a*) prompted us to prospectively determine the clinical significance of MDR1 gene expression in colorectal carcinomas. One objective was to evaluate both frequency and intensity of MDR1 gene expression on a large study population in order to assess whether MDR1 gene expression might be considered as one of the mechanisms involved in the drug resistance of these tumours, which often are intrinsically resistant to cytotoxic drugs (Cohen *et al.*, 1989). Another major goal of our study was to assess the association of MDR1 RNA transcripts with other clinical parameters. In particular, we wanted to determine the relationship between MDR1 gene expression of the carcinomas and the durations of both disease-free and overall survival of the patients in order to determine whether the multidrug resistance phenotype is associated with a more aggressive disease. The possibility of such an association was previously raised by Weinstein *et al.* (1990; 1991) who found that P-glycoprotein expression at the leading edge of the tumours was associated with local tumour aggressiveness and lymph node metastasis but did not report whether this association also translated into different survival durations of the patients. The results of our study are reported here.

### Patients and methods

### *Tumour specimens*

From 1988 to 1991, specimens of colorectal carcinomas were obtained from 113 patients (58 females, 55 males) who underwent surgery at the General Hospital of Wr. Neustadt, Austria. Tumour specimens were immediately frozen and stored at − 70°C until use.

### *Cell lines*

Drug-sensitive KB-3-1 cells and multidrug-resistant KB-8-5 cells (provided by Dr I. Pastan, NIH, Bethesda, MD, USA) were grown as described (Pirker *et al.*, 1991; Wallner *et al.*, 1991).

### *Determination of MDR1 gene expression*

MDR1 RNA levels of tumour specimens and cell lines were determined by a slot blot technique as described in detail elsewhere (Pirker *et al.*, 1991; Wallner *et al.*, 1991). Briefly, tumour specimens were homogenised in RNAzol (Cinna/Biotecx Lab. Int. Inc., Friendswood, Texas). Total RNA was extracted by means of RNAzol as described in the manufacturer's manual. The intactness of the RNA was confirmed by agarose formaldehyde gel electrophoresis. RNA was blotted onto nylon membrane filters. After prehybridisation, filters were hybridised with a radio-labelled MDR1 cDNA (probe 5A; provided by Dr Ira Pastan and Dr Michael Gottesman, National Cancer Institute, Bethesda, MD, USA) in 50% formamide, 5 × SSC, 20 mM sodium phosphate buffer (pH 6.5), 10% dextran sulfate, 1 × Denhardt's Reagent and 0.2 mg $ml^{-1}$ salmon sperm DNA at 42°C for 18–20 h. After washings, auto-radiographic exposures lasted for 2–5 days (Figure 1). RNA loading was normalised to actin expression (Pirker *et al.*, 1991). No expression was seen in drug-sensitive KB-3-1 cells and an arbitrary value of 30 units (U) was assigned to the MDR1 RNA expression of 10 µg of total RNA from multidrug-resistant KB-8-5 cells (Goldstein *et al.*, 1989; Pirker *et al.*, 1991).

### *Determination of expression of MDR2 RNA*

The MDR2 RNA levels were determined by a slot blot hybridisation, using a human MDR2 (MDR3) probe (40mer, Pr-1, Oncogene Science), which had been radiolabelled with gamma- $^{32}$P-ATP. Filters were prehybridised overnight at 65°C in 1 M NaCl, 50 mM Tris/HCl buffer (pH 7.5), 5 × Denhardt's Reagent, 1% SDS and salmon sperm DNA. Hybridisation was then performed overnight at 65°C in 1 M NaCl, 50 mM Tris/HCl buffer (pH 7.5), 5 × Denhardt's Reagent, 1% SDS, 10% dextran sulfate and salmon sperm DNA. After washings with 2 × SSC containing 0.1% SDS (5 min at room temperature; 30 min at 65°C; 5 min at room temperature), autoradiography was performed.

### *Survival analysis*

Durations of disease-free survival and of overall survival were estimated according to Kaplan-Meier (1958) in 91 and 103 patients, respectively. Among these evaluable patients, 30 patients with tumour stages II or III, participating in an adjuvant chemotherapy trial, received postoperative intravenous chemotherapy with 5-fluorouracil and leucovorin over a period of 6 months, supplemented with intraperitoneal chemotherapy in some cases. The remaining patients had no further treatment after surgery.

Correspondence: R. Pirker, Clinic for Internal Medicine I, Department of Oncology, Währingergürtel 18–20, A-1090 Vienna, Austria.
Received 10 December; and in revised form 23 May 1993.

**Figure 1** Slot blot analysis for MDR1 RNA. MDR1 RNA expression (left lane) was determined by slot blot analysis. Actin expression (right lane) was used to ensure RNA loading and to correct for different amounts of RNA loaded. MDR1 RNA levels were negative in drug-sensitive KB-3-1 cells (10 μg RNA; negative control) and positive in multidrug-resistant KB-8-5 cells (10, 3 and 1 μg RNA; positive control). The slot at the bottom refers to a colorectal tumour specimen.

### *Statistical analysis*

Frequencies were tested by chi-square tests. Comparisons of survival durations between patients with MDR1 RNA positive tumours and those with MDR1 RNA negative tumours were done with the Wilcoxon test.

### Results

MDR1 RNA expression of 113 primary colorectal carcinomas was determined by slot blot analysis and compared with the expression of drug-sensitive KB-3-1 cells and 4- to 6-fold multidrug-resistant KB-8-5 cells (Figure 1). MDR1 RNA levels were negative in 40 (35%) and positive in 73 (65%) carcinoma samples (Table I). MDR1 RNA levels were low, intermediate and high (>9 U) in 32 (28%), 26 (23%) and 15 (13%) tumour specimens, respectively. To exclude the possibility that the observed MDR1 transcripts were due to the expression of the MDR2 gene, which *in vitro* does not correlate with the degree of multidrug resistance (Pastan & Gottesman, 1987), we also measured the MDR2 RNA levels in 98 tumours. However, no expression of MDR2 RNA was detected (Table I).

The clinical data of the patients are summarised in Table II. Histological examination revealed adenocarcinomas in all cases. MDR1 gene expression of the tumours was independent of age and sex of the patients, localisation and size of the primary tumour, tumour infiltration of the lymph nodes, distant metastases and histologic grading (Table II).

To evaluate whether MDR1 gene expression has any impact on the clinical course of colorectal carcinomas, Kaplan-Meier analysis of disease-free survival and of overall survival was performed in 91 and 103 patients, respectively. The median duration of follow-up of the patients was 2 years and was not different between patients with MDR1 RNA positive tumours and those with negative tumours (data not shown). With respect to the total study population, the durations of disease-free survival (Figure 2) and of overall survival (Figure 3) were independent of MDR1 RNA expression. Both among patients with tumour stage II and among patients with tumour stage III, MDR1 RNA expression had no impact on the durations of survival (data not shown).

**Table I** Expression of the MDR1 and MDR2 genes in primary colorectal carcinomas

| | *No. (%)* |
|---|---|
| *MDR1 gene* | |
| Evaluable tumours | 113 (100%) |
| MDR1 RNA negative tumours | 40 (35%) |
| MDR1 RNA positive tumours | 73 (65%) |
| low | 32 (28%) |
| intermediate | 26 (23%) |
| high (>9 U) | 15 (13%) |
| *MDR2 gene* | |
| Evaluable tumours | 98 (100%) |
| MDR2 RNA negative tumours | 98 (100%) |
| MDR2 RNA positive tumours | 0 (0%) |

MDR1 and MDR2 RNA expression of primary colorectal carcinomas were determined by slot blot analysis.

**Table II** MDR1 RNA levels and clinical data of the patients

| | *all pts.* | *MDR1 negat. pts.* | *MDR1 pos. pts.* | *P-value* |
|---|---|---|---|---|
| *Number of pts.* | 113 | 40 | 73 | |
| *Age (yrs)* | | | | |
| Median | 60 | 61 | 59 | NS |
| Range | 34–86 | 40–83 | 34–86 | |
| *Sex (f/m)* | 58/55 | 20/20 | 38/35 | |
| *Localisation of the primary tumour* | | | | |
| Colon | 61% | 57% | 64% | NS |
| Rectum | 38% | 43% | 36% | |
| *Histologic grade* | | | | |
| G1 | 23% | 11% | 26.5% | |
| G2 | 72% | 89% | 66.5% | NS |
| G3 | 5% | – | 7% | |
| *Primary tumour* | | | | |
| T1 | 1% | 2% | – | |
| T2 | 39% | 43% | 37% | |
| T3 | 32% | 23% | 37% | NS |
| T4 | 22% | 27% | 19% | |
| TX | 6% | 5% | 7% | |
| *Regional lymph nodes* | | | | |
| N0 | 60% | 63% | 60% | |
| N1 | 29% | 33% | 28% | |
| N2 | 4% | – | 5% | NS |
| N3 | 1% | 2% | – | |
| NX | 6% | 2% | 7% | |
| *Distant metastasis* | | | | |
| M0 | 82% | 88% | 79% | |
| M1 | 12% | 7% | 14% | NS |
| MX | 6% | 5% | 7% | |
| *Patients (%) with adjuvant chemotherapy* | 27% | 18% | 32% | |

**Figure 2** MDR1 RNA expression of colorectal carcinomas and disease-free survival of the patients. The duration of disease-free survival was determined according to Kaplan-Meier in 91 patients. The survival duration was not different between patients with MDR1 RNA positive tumours and those with negative tumours.

**Figure 3** MDR1 RNA expression of colorectal carcinomas and overall survival of the patients. The duration of overall survival of the patients was determined according to Kaplan-Meier in 103 patients. MDR1 RNA expression did not affect the duration of the overall survival.

When patients with rectal carcinomas and those with colon carcinomas were separately analysed, also no association between MDR1 RNA expression and survival durations was observed (data not shown). Since the percentage of patients receiving adjuvant chemotherapy was similar for patients with MDR1 RNA negative tumours as compared to those with positive tumours (Table II), it is unlikely that our results were affected by adjuvant chemotherapy. Nevertheless, we also separately analysed patients with adjuvant chemotherapy and those without adjuvant chemotherapy. However, in both groups the durations of disease-free as well as overall survival of the patients were independent of MDR1 RNA expression of the tumours (data not shown).

### Discussion

Our prospective study demonstrated the expression of the MDR1 gene in 65% of the primary colorectal carcinomas examined and thus confirms previous reports on MDR1 gene expression in these tumours (Pastan & Gottesman, 1987; Goldstein *et al.*, 1989). We have determined MDR1 gene expression by measuring MDR1 RNA levels of the tumours, using a sensitive and semiquantitative slot blot analysis (Goldstein *et al.*, 1989; Pirker *et al.*, 1991; Wallner *et al.*, 1991). Because MDR2 RNA expression was not detected in the tumours, the observed expression of the MDR1 gene did not result from cross-reactivity of the MDR1 probe with transcripts of the MDR2 gene. Alternatively, immunohistochemical detection of P-glycoprotein can be applied in order to demonstrate the expression of the MDR1 gene in tissues (Thiebaut *et al.*, 1987; Weinstein *et al.*, 1991; Chan *et al.*, 1990; Wishart *et al.*, 1990). Recently, expression of P-glycoprotein was seen in 65 out of 95 primary colon carcinomas (Weinstein *et al.*, 1991), which is consistent with the percentage of MDR1 RNA positive tumours in our study. Whether MDR1 RNA analysis or immunohistochemical detection of P-glycoprotein is better suited for clinical purposes, remains to be determined.

The expression of the MDR1 gene in normal colon epithelium (Thiebaut *et al.*, 1987; Fojo *et al.*, 1987*a*) suggests that the MDR1 gene might be expressed predominantly in well differentiated tumours. However, we did not find a correlation between histologic grade and MDR1 transcripts of the carcinomas. MDR1 RNA expression was also independent of localisation and size of the primary tumour. These findings are consistent with the study by Weinstein *et al.* (1991) who found that expression of P-glycoprotein in colorectal carcinomas was independent of tumour size and histologic grade. Weinstein *et al.* (1991), however, reported a higher incidence of lymph node metastasis when P-glycoprotein positive cells were present at the leading edge of the primary tumour, suggesting that expression of P-glycoprotein is associated with local tumour aggressiveness. In contrast, we found no association between MDR1 RNA expression of the primary tumour and tumour infiltration of the lymph nodes or distant metastases (Table II). It should be noted, however, that we have evaluated the MDR1 RNA levels of the tumour specimens as a total and not only of specific areas within the specimens.

Our results indicate that multidrug-resistant cells are present in the carcinomas prior to any chemotherapy. The high percentage of MDR1 RNA positive colorectal carcinomas could, at least partly, account for the well known intrinsic resistance of these tumours to anthracyclines and other drugs transported by P-glycoprotein. MDR1 gene expression is assumed to be involved also in the clinical drug resistance of other malignant diseases (Bell *et al.*, 1985; Gerlach *et al.*, 1987; Fojo *et al.*, 1987*b*; Dalton *et al.*, 1989; Goldstein *et al.*, 1989; Pirker *et al.*, 1989; Chan *et al.*, 1990; Wishart *et al.*, 1990; Pirker *et al.*, 1991; Wallner *et al.*, 1991, 1993). In neuroblastomas (Chan *et al.*, 1991), soft tissue sarcomas of childhood (Chan *et al.*, 1990) and adult acute myeloid leukemias (Pirker *et al.*, 1991; Marie *et al.*, 1991; Campos *et al.*, 1992), MDR1 gene expression of the malignant cells was associated with shorter survival durations of the patients who had been treated with protocols that included anthracyclines. In the present study, we did not observe an impact of MDR1 gene expression of the tumours on the durations of disease-free survival and overall survival of the patients (Figures 2 and 3), indicating that MDR1 RNA expression of tumour specimens is of no value for estimating prognosis. Whether P-glycoprotein expression at the leading edge of the tumours, a situation probably associated with a more aggressive disease (Weinstein *et al.*, 1991), might predict prognosis remains to be determined. The lack of an association of MDR1 RNA expression with clinical outcome in our study can be explained by several reasons. Firstly, the biological growth behaviour of MDR1 RNA positive tumours might in fact not be different from their negative counterparts. Secondly, our patients were not treated with drugs that are affected by the MDR1 gene. Response to 5-fluorouracil and leucovorin should be independent of a functionally active MDR1 gene because both drugs are not transported by P-glycoprotein. Thirdly, we cannot exclude that the applied slot blot technique contributed to the observed lack. Finally, longer follow-up of our patients might demonstrate some impact of MDR1 RNA expression of the tumours on the long-term survival of the patients.

Future studies will also have to address the impact of MDR1 gene expression (of the primary or metastatic tumour) on the resistance of the metastatic tumours to drugs that are pumped by P-glycoprotein. If such an association can be demonstrated, clinical trials with resistance modifiers (Dalton *et al.*, 1989; Pirker *et al.*, 1990; Twentyman 1992) might be warranted in patients with metastatic colorectal carcinomas.

R. Pirker has been supported by the 'Fonds zur Förderung der wissenschaftlichen Forschung' (Project P8752).

### References

BELL, D.R., GERLACH, J.H., KARTNER, N., BUICK, R.N. & LING, V. (1985). Detection of P-glycoprotein in ovarian cancer: a molecular marker associated with multidrug resistance. *J. Clin. Oncol.*, **3**, 311–315.

CAMPOS, L., GUYOTAT, D., ARCHIMBAUD, E., CALMARD-ORIOL, P., TSURUO, T., TRONCY, J., TREILLE, D. & FIERE, D. (1992). Clinical significance of multidrug resistance P-glycoprotein expression on acute nonlymphoblastic leukemia cells at diagnosis. *Blood*, **79**, 473–476.

CHAN, H.S.L., THORNER, P.S., HADDAD, G. & LING, V. (1990). Immunohistochemical detection of P-glycoprotein: prognostic correlation in soft tissue sarcoma of childhood. *J. Clin. Oncol.*, **8**, 689–704.

CHAN, H.S.L., HADDAD, G., THORNER, P.S., DEBOER, G., PING LIN, Y., ONDRUSEK, N., YEGER, H. & LING, V. (1991). P-glycoprotein expression as a predictor of the outcome of therapy for neuroblastoma. *N. Engl. J. Med.*, **325**, 1608–1614.

COHEN, A.M., SHANK, B. & FRIEDMAN, M.A. (1989). Colorectal cancer. In *Cancer: Principles and Practice of Oncology*, DeVita, V.T., Jr, Hellman, S., Rosenberg, S.A. (eds). pp. 895–964. Lippincott: Philadelphia.

DALTON, W.S., GROGAN, T.M., MELTZER, P.S., SCHEPER, R.J., DURIE, B.G.M., TAYLOR, C.W., MILLER, T.P. & SALMON, S.E. (1989). Drug-resistance in multiple myeloma and non-Hodgkin's lymphoma: detection of P-glycoprotein and potential circumvention by addition of verapamil to chemotherapy. *J. Clin. Oncol.*, **7**, 415–424.

FOJO, A.T., UEDA, K., SLAMON, D.J., POPLACK, D.G., GOTTESMAN, M.M. & PASTAN, I. (1987*a*). Expression of a multidrug-resistance gene in human tumors and tissues. *Proc. Natl Acad. Sci. USA*, **84**, 265–269.

FOJO, A.T., SHEN, D.W., MICKLEY, L.A., PASTAN, I. & GOTTESMAN, M.M. (1987*b*). Intrinsic drug resistance in human kidney cancer is associated with expression of a human multidrug-resistance gene. *J. Clin. Oncol.*, **5**, 1922–1927.

GERLACH, J.H., BELL, D.R., KARAKOUSIS, C., SLOCUM, H.K., KARTNER, N., RUSTUM, Y.M., LING, V. & BAKER, R.M. (1987). P-glycoprotein in human sarcoma: evidence for multidrug resistance. *J. Clin. Oncol.*, **5**, 1452–1460.

GOLDSTEIN, L.J., GALSKI, H., FOJO, A., WILLINGHAM, M., LAI, S., GAZDAR, A., PIRKER, R., GREEN, A., CRIST, W., BRODEUR, G.M., GRANT, C., LIEBER, M., COSSMAN, J., GOTTESMAN, M.M. & PASTAN, I. (1989). Expression of a multidrug resistance gene in human tumors. *J. Natl Cancer Inst.*, **81**, 116–124.

GOTTESMAN, M.M. & PASTAN, I. (1988). The multidrug transporter, a double-edged sword. *J. Biol. Chem.*, **263**, 12163-12166.

KAPLAN, E.L. & MEIER, P. (1958). Nonparametric estimation from incomplete observations. *J. Am. Stat. Assoc.*, **53**, 457–481.

MARIE, J.P., ZITTOUN, R. & SIKIC, B.I. (1991). Multidrug resistance (mdr1) gene expression in adult acute leukemias: correlations with treatment outcome and *in vitro* drug sensitivity. *Blood*, **78**, 586–592.

PASTAN, I. & GOTTESMAN, M.M. (1987). Multiple-drug resistance in human cancer. *New Engl. J. Med.*, **316**, 1388–1393.

PIRKER, R., GOLDSTEIN, L.J., LUDWIG, H., LINKESCH, W., LECHNER, C., GOTTESMAN, M.M. & PASTAN, I. (1989). Expression of a multidrug resistance gene in blast crisis of chronic myelogeneous leukemia. *Cancer Communications*, **1**, 141–144.

PIRKER, R., KEILHAUER, G., RASCHACK, M., LECHNER, C. & LUDWIG, H. (1990). Reversal of multidrug resistance in human KB cell lines by structural analogs of verapamil. *Int. J. Cancer*, **45**, 916–919.

PIRKER, R., WALLNER, J., GEISSLER, K., LINKESCH, W., HAAS, O.A., BETTELHEIM, P., HOPFNER, M., SCHERRER, R., VALENT, P., HAVELEC, L., LUDWIG, H. & LECHNER, K. (1991). MDR1 gene expression and treatment outcome in acute myeloid leukemia. *J. Natl Cancer Inst.*, **83**, 708–712.

RIORDAN, J.R., DEUCHARS, K., KARTNER, N., ALON, N., TRENT, J. & LING, V. (1985). Amplification of P-glycoprotein genes in multidrug-resistant mammalian cell lines. *Nature*, **316**, 817–819.

RONINSON, I.B., CHIN, J.E., CHOI, K., GROS, P., HOUSMAN, D., FOJO, A., SHEN, D.W., GOTTESMAN, M.M. & PASTAN, I. (1986). Isolation of human mdr DNA sequences amplified in multidrug-resistant KB carcinoma cells. *Proc. Natl Acad. Sci. USA*, **83**, 4538–4542.

THIEBAUT, F., TSURUO, T., HAMADA, H., GOTTESMAN, M.M., PASTAN, I. & WILLINGHAM, M.C. (1987). Cellular localization of the multidrug-resistance gene product P-glycoprotein in normal human tissues. *Proc. Natl Acad. Sci. USA*, **84**, 7735–7738.

TWENTYMAN, P.R. (1992). MDR1 (P-glycoprotein) gene expression: implications for resistance modifier trials. *J. Natl Cancer Inst.*, **84**, 1458–1460.

WALLNER, J., DEPISCH, D., HOPFNER, M., HAIDER, K., SPONA, J., LUDWIG, H. & PIRKER, R. (1991). MDR1 gene expression and prognostic factors in primary breast carcinomas. *Eur. J. Cancer*, **27**, 1353–1357.

WALLNER, J., DEPISCH, D., GSUR, A., GÖTZL, M., HAIDER, K. & PIRKER, R. (1993). MDR1 gene expression and its clinical relevance in primary gastric carcinomas. *Cancer*, **71**, 667–671.

WEINSTEIN, R.S., KUSZAK, J.R., KLUSENS, L.F. & COON, J.S. (1990). P-glycoprotein in pathology: The multidrug resistance gene family in humans. *Human Pathol.*, **21**, 34–48.

WEINSTEIN, R.S., JAKATE, S.M., DOMINGUEZ, J.M., LEBOVITZ, M.D., KOUKOULIS, G.K., KUSZAK, J.R., KLUSENS, L.F., GROGAN, T.M., SACLARIDES, T.J., RONINSON, I.B. & COON, J.S. (1991). Relationship of the expression of the multidrug resistance gene product (P-glycoprotein) in human colon carcinoma to local tumor aggressiveness and lymph node metastatis. *Cancer Res.*, **51**, 2720–2726.

WISHART, G.C., PLUMB, J.A., GOING, J.J., MCNICOL, A.M., MCARDLE, C.S., TSURUO, T. & KAYE, S.B. (1990). P-glycoprotein expression in primary breast cancer detected by immunocytochemistry with two monoclonal antibodies. *Br. J. Cancer*, **62**, 758–761.